# Chimeric RNA Design Principles for RNA-Mediated Gene Fusion

**DOI:** 10.3390/cells11061002

**Published:** 2022-03-16

**Authors:** Sachin Kumar Gupta, Laising Yen

**Affiliations:** 1 Department of Pathology & Immunology, Baylor College of Medicine, Houston, TX 77030, USA; sachinkumar.gupta@bcm.edu; 2 Department of Molecular & Cellular Biology, Baylor College of Medicine, Houston, TX 77030, USA; 3 Dan L. Duncan Cancer Center, Baylor College of Medicine, Houston, TX 77030, USA

**Keywords:** TMPRSS2-ERG, chimeric RNA, gene fusion, genomic recombination, prostate cancer

## Abstract

One common genetic alteration in cancer is gene fusion resulting from chromosomal translocations. The mechanisms that create such oncogenic fusion genes are not well understood. Previously, we provided the direct evidence that expression of a designed chimeric RNA can drive the formation of TMPRSS2-ERG gene fusion. Central to this RNA-mediated gene fusion mechanism is a proposed three-way junction formed by RNA/DNA hybrid and the intergenic DNA stem formed by target genes. In this study, we determined the important parameters for chimeric RNA-mediated gene fusion using TMPRSS2-ERG fusion gene as the model. Our results indicate that both the chimeric RNA lengths and the sizes of unpaired bulges play important roles in inducing TMPRSS2-ERG gene fusion. The optimal length of unpaired bulges was about 35 nt, while the optimal chimeric RNA length was about 50 nt for targeting. These observations were consistent regardless of the target locations within TMPRSS2 and ERG genes. These empirically determined parameters provide important insight for searching cellular RNAs that may initiate oncogenic fusion genes. The knowledge could also facilitate the development of useful genomic technology for manipulating mammalian genomes.

## 1. Introduction

Gene fusion is one of the most important chromosomal alterations in cancer [1]. In prostate cancer, the oncogenic fusion gene TMPRSS2-ERG resulting from chromosomal translocations is present in 50% of the patient population [2]. It leads to androgen-dependent overexpression of ERG, which increases cell invasion and proliferation [3,4,5]. The mechanisms that create such oncogenic fusion genes remain poorly understood. In our previous study, we reported an unappreciated RNA-driven mechanism in which the expression of a short designer RNA with a chimeric sequence resembling that of TMPRSS2 and ERG genes leads to TMPRSS2-ERG gene fusion in prostate cells [6]. The process is specified by the sequence of chimeric RNA involved, and facilitated by DHT (dihydrotestosterone), a testosterone hormone analog. In addition, it is the antisense rather than sense chimeric RNAs that effectively drive gene fusion. Importantly, such an RNA-driven gene fusion is not a mechanism restricted only to human prostate cells. Recently [7], we provided evidence that expression of a designer chimeric RNA targeting JAZF1 and SUZ12 genes in human endometrial stromal cells also drives the formation of JAZF1-SUZ12, a cancer fusion gene commonly found in low-grade endometrial stromal sarcomas patients [8,9,10].

**Figure 1:**
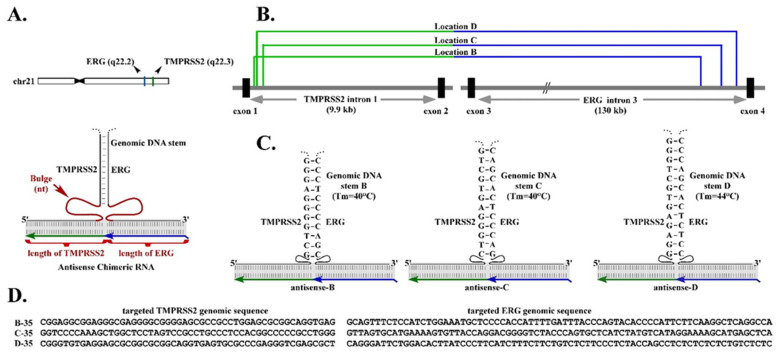
A model of three-way junction formation in RNA-mediated TMPRSS2-ERG gene fusion. (**A**) Upper panel: chromosomal locations of TMPRSS2 and ERG genes. Both TMPRSS2 and ERG genes are on the minus strand of chromosome 21, separated by 3 Mb, an intra-chromosomal configuration prone to rearrangements. Lower panel: schematic illustration of a three-way junction formed between genomic DNA and chimeric RNA. The three-way junction model consists of the RNA/DNA duplex and the intergenic DNA stem formed by the genomic TMPRSS2 sequence complementary to the genomic ERG sequence. Our study highlights the effects of two essential elements in the proposed three-way junction: (1) the unpaired bulges linking between the RNA/DNA duplex and the intergenic DNA stem and (2) the chimeric RNA length for forming the RNA/DNA duplex. Both elements are shown in red. (**B**) Three independent target locations used in our previous study where the designer chimeric RNAs are known to induce TMPRSS2-ERG gene fusion. As these locations are in the introns, the designed chimeric RNAs targeting them contain only intronic sequences and no exonic sequences. (**C**) The putative three-way junction formed between the targeted genomic DNA locations (black) and the designed antisense chimeric RNAs (green/blue). An intergenic DNA stem can occur when the TMPRSS2 sequence is complementary to the ERG sequence near the junction site. The intergenic DNA stem may include a high-energy G·T and A·C wobble-pair known to have Watson–Crick-like geometry in a DNA double helix. (**D**) Examples of genomic sequences targeted by antisense chimeric RNA asB-35, asC-35, and as-D35. The targeted sequences contained a 75-nt ERG gene and a 52-nt TMPRSS2 gene. In these cases, a bulge of 35 nt will be created when the chimeric RNAs form an RNA/DNA duplex with the genomic sequences.

These results, derived from two independent cases of RNA-driven gene fusion, support a model where the chimeric RNA sequence invades the target genes to stabilize a transient RNA/DNA duplex reminiscent of R-loops [11,12,13,14,15]. Resolving such an RNA/DNA duplex by DNA break/repair mechanisms yields the final gene fusion through recombination in regions prone to DNA breaks. One fundamental observation in our previous studies was that the gene fusion process is specified by the sequence of the chimeric RNA involved [6,7]. For example, in prostate cells, the chimeric RNA targeting TMPRSS2 and ERG genes induced TMPRSS2-ERG gene fusion but not TMPRSS2-ETV1 gene fusion. Conversely, targeting TMPRSS2 and ETV1 genes by specific chimeric RNA induced TMPRSS2-ETV1 gene fusion but not TMPRSS2-ERG gene fusion [6]. Furthermore, over-expression of RNase-H, which degrades the RNA in an RNA/DNA duplex, significantly reduced the efficiency of chimeric RNA-induced gene fusion [6]. Together, they indicate that chimeric RNA mediates genome rearrangements by forming an RNA/DNA duplex through ‘base-pairing’ with target genes (Figure 1A). However, the optimal length of chimeric RNA required to form an effective RNA/DNA duplex with the two parental genes is yet to be determined.

A second fundamental observation derived from our previous studies is that, in addition to the RNA/DNA duplex, an intergenic DNA stem that could be formed by the genomic TMPRSS2 sequence paired with the genomic ERG sequence, may play a role in RNA-mediated TMPRSS2-ERG gene fusion [6] (Figure 1A). This observation was derived from the results that chimeric RNAs targeting genomic regions of TMPRSS2 and ERG that can form stable intergenic DNA stems efficiently induce gene fusion. In contrast, targeting regions with unstable intergenic DNA stems (that have lower Tm) resulted in no gene fusion induction [6]. Together, the results suggest that a higher-order structural motif resembling a three-way junction consisting of the RNA/DNA duplex and intergenic DNA stem may be necessary for efficient RNA-mediated gene fusion.

A three-way junction is a structural motif commonly found in naturally occurring RNA molecules such as ribosomal RNAs, which contain junction structures ranging from three-way to seven-way junctions, and most of which include stretches of unpaired nucleotides called “bulges” at the branch point [16,17]. These bulges provide flexible hinges in the higher-order structures, as the unpaired residue is not restricted by base-pairing interactions. Bulges are known to facilitate the coaxial stacking of the flanking stems and stabilize nucleic acid three-way junctions [18,19]. Our earlier study indicated that moving the chimeric RNA target regions, therefore alternating the sizes of bulge linking between the RNA/DNA duplex and the proposed intergenic DNA stem, greatly influences the efficiency of RNA-mediated gene fusion [6]. Yet, the optimal size of bulge for inducing gene fusion is yet to be determined.

In this report, we present the empirical data that determines the important parameters—the optimal chimeric RNA length for forming RNA/DNA duplex, and the optimal bulge size for efficient RNA-mediated gene fusion. Studying these parameters is important, as they provide the design principles for developing new technology for manipulating the mammalian genome through large-distance DNA rearrangements. Secondly, these parameters provide valuable bioinformatics guidelines for searching for cellular chimeric-like RNAs that have the potential of inducing oncogenic fusion genes. Lastly, this knowledge could facilitate the development of therapeutic strategies to inhibit the formation of RNA/DNA duplexes and three-way junctions, therefore preventing the formation of cancer fusion genes and future tumors.

## 2. Materials and Methods

### 2.1. LNCaP Cell Culture

LNCaP cells are epithelial cells derived from a human prostate carcinoma. For transient transfection experiments, LNCaP cells were routinely cultured in RPMI 1640 medium (RPM1 1640, 1X, with L-glutamine, #10-040-CV, CORNING cellgro Manassas, VA, USA) containing 10% fetal bovine serum (premium grade FBS, #1500-500, VWR Life Sciences, Radnor, PA, USA) and 1% penicillin/streptomycin (#15140-122, Gibco Fisher Scientific, Hampton, NH, USA) in a 5% CO_2_ humidified incubator.

### 2.2. Plasmid Constructions

The chimeric RNAs were expressed using an expression plasmid with a human U6 promoter, a pol-III promoter. The human U6 promoter was constructed by cloning the region (−718 to +40) of the human U6 gene ‘RNU6-1′ from the genomic DNA of HEK-293T cells. This genomic segment contains a sequence upstream of the U6 promoter, the transcription start (+1), and a 40-nt stem loop cap sequence, followed by added multiple cloning sites (Pst-I and Hind-III). The chimeric RNA sequences were designed using target intron sequences of ERG and TMPRSS2 in antisense orientation (see Supplementary Materials). They were generated by PCR using forward primers containing a Pst-I restriction site and a reverse primer carrying the U6 transcription termination signal “TTTTTT” and Hind-III restriction site. To eliminate potential transcription pre-termination, any stretch of four to six ‘T’s in chimeric RNA sequence was mutated to carry one ‘A’ in the middle. For example, ‘TTTTTT’ was mutated to TTATTT’, ‘TTTTT’ was mutated to ‘TTATT, and ‘TTTT’ was mutated to ‘TATT’.

### 2.3. Transient Transfection of Plasmids for Chimeric RNA Expression

Twenty hours prior to transfection, LNCaP cells were seeded in 12-well plate (BioLite 12 Well Multidish, #130185, Thermo Fisher Scientific, Waltham, MA, USA) with a density of 5 × 10^5^ cells/well and 1 mL/well of culture medium, as described above. Transfection was performed using Turbofect transfection reagent (Thermo Scientific, #R0531) according to manufacturer’s protocol. Briefly, 1 µg of a particular plasmid was first diluted in 100 µL of the serum-free DMEM followed by immediate mixing by pipetting. Then, 4 µL of the transfection reagent was added to the diluted DNA followed by mixing and incubation for 20 min. The DNA/transfection reagent mixture was then added drop-wise to a well containing LNCaP cells in 1 mL medium. Cells were then incubated in a CO_2_ humidified incubator at 37 °C for 72 h for the expression of the chimeric RNAs.

### 2.4. DHT Preparation and Treatment

DHT (dihydrotestosterone) was purchased from Sigma Aldrich (5α-Androstan-17β-ol-3-one, #A8380). Concentrated stock of 1500 µM was prepared by dissolving 4.3566 mg of DHT powder in 10 mL of 100% ethanol (200-proof ethanol, Koptec, king of prussia, PA, USA. #V1016) and then aliquoted in 1 mL tubes and stored at −80 °C. For treating cultured cells, concentrated DHT stock was diluted as 10× working solutions (for example, for 0.9 µM final concentration, 10× was prepared as 9.0 µM) with the appropriate complete culture medium and used immediately.

### 2.5. RNA Isolation from Cells

Total RNA from cultured cells was extracted using a Ribopure Kit according to the manufacturer’s instructions (#AM1924, Invitrogen Waltham, MA, USA). Briefly, cells were homogenized/lysed in 1 mL TRI reagent followed by 5 min incubation at room temperature. This incubation allowed nucleoprotein complexes to dissociate completely. Then, 200 µL of chloroform was added followed by vortexing at maximum speed for 15 s. The mixture was then incubated at room temperature for 10 min. The lysate was then centrifuged at 12,000× *g* for 10 min at 4 °C to separate the mixture into a lower organic phase; an interphase; and an upper, aqueous phase. RNA remained in the aqueous phase while DNA and proteins were in the interphase and organic phase. Then, 400 µL of the upper aqueous phase was extracted in a new tube and 200 µL of 100% ethanol was added followed by immediate vortexing at maximum speed for 5 s to avoid RNA precipitation. The sample was then passed through the filter assembly resulting in the binding of the nucleic acids to the filter. The column was then rinsed twice with wash buffer and total RNA was then eluted in a new tube for further analysis. For detection of residual genomic and plasmid DNA, eluted RNA was subject to PCR reaction with primers specific to intron regions of the house-keeping gene GAPDH, and with primers specific to the transfected plasmid. Total RNA was converted to cDNA only if it is validated as free of DNA contamination.

### 2.6. Reverse Transcription Reaction

Next, 1 µg of total RNA was used for each reverse transcription reaction according to manufacturer’s instruction (superscript III RT, # 18080-051, Invitrogen). RNA was converted to cDNA with oligo dT primer. After the addition of dNTPs, the mixture was denatured at 65 °C for 5 min. This was followed by the addition of a master-mix containing 1× superscript buffer, 10 mM DTT, 5 mM magnesium chloride, RNaseOUT, and SuperScript III reverse transcriptase. Reactions were carried out at 50 °C for 50 min and then terminated by incubation at 85 °C for 5 min. cDNA was then treated with RNase-H for 20 min at 37 °C to degrade RNA in the DNA/RNA hybrid and 1 µL of cDNA was used as template for each subsequent PCR reaction.

### 2.7. RT-PCR for Detecting Induced Fusion Transcripts

The induced TMPRSS2-ERG fusion RNA and control GAPDH RNA were detected using one-round RT-PCR. PCR was done with a standard three-step protocol using RED-Taq DNA polymerase (#D5684-1KU, Sigma St. Louis, MO, USA) according to the manufacturer’s instruction. The annealing temperature for both TMPRRS2-ERG and GAPDH primers was 57 °C. RT-PCR primers for amplifying induced fusion RNA TMPRSS2-ERG were TMPRSS2 ex-1 F1: 5′-TAGGCGCGAGCTAAGCAGGAG-3′ and ERG ex-4 R1: 5′-CTTGAGCCATTCACCTGGCTAG-3′. RT-PCR primers for amplifying the GAPDH RNA were GAPDH F1: 5′-GCGTCTTCACCACCATGGAGA-3′ and GAPDH R1: 5′-AGCCTTGGCAGCGCCAGTAGA-3′.

### 2.8. The Melting Temperature (Tm)

Tm was calculated using the formula: Tm = 64.9 + 41 * (nG + nC − 16.4)/(nA + nT + nG + nC) where “n” stands for total number of particular nucleotide.

### 2.9. Quantitation of RT-PCR

Each RT-PCR experiment was repeated three times. GAPDH was used as internal control for the amount of RNA loaded. The band intensities were quantified using ImageJ 1.5.3. In brief, band intensity was calculated for each individual band using ImageJ. Background intensity was subtracted using the averaged background noise obtained from two areas—one from above and one below the band. The corrected band intensity was then normalized to the band intensity of GAPDH. Experiments were repeated thrice and the mean and standard deviation were calculated and presented using Microsoft Office 2016 Excel.

## 3. Results

### 3.1. Bulge Size Regulates the Efficiency of RNA-Mediated Gene Fusion

Previously we demonstrated that the expression of a designer chimeric RNA can lead to the induction of TMPRSS2-ERG gene fusion in prostate cells [6]. Among all chimeric RNAs that we designed, ‘antisense-5′ ranks as the most potent chimeric RNA. The proposed three-way junction model, consisting of the RNA/DNA duplex formed by antisense-5 and the intergenic DNA stem formed by TMPRSS2 and ERG genes, suggests an unpaired bulge of 36 nt on the TMPRSS2 side and 47 nt on the ERG side [6]. This raises the question of whether a bulge size of 36–47 nt is necessary or optimal for fusion gene induction. To answer this question, we selected three independent target locations (Figure 1B) used in our previous study where the designer chimeric RNAs are known to induce TMPRSS2-ERG gene fusion [6]. Figure 1C illustrates the three-way junction models formed by these designer chimeric RNAs (antisense-B, -C, and-D) at those locations, and the corresponding intergenic DNA stems forged by them (stem B, C, and D). Because these are different locations in the introns of TMPRSS2 and ERG genes (Figure 1B), the chimeric RNAs targeting them have completely different sequences. An example of targeted sequences at each location is shown in Figure 1D. In addition, the intergenic DNA stems forged by these chimeric RNA are also composed of different and unrelated sequences. Therefore, the three selected locations represent three independent experimental examples for which the optimal bulge size can be determined independently.

To determine the optimal bulge size, we designed three series of antisense chimeric RNAs, with each series targeting locations B, C, or D. Each series of chimeric RNAs was designed to test ten different bulge sizes: 2, 16, 20, 25, 31, 35, 40, 45, 50 and 100 nt while maintaining the same intergenic DNA stem. To create different bulge sizes, the chimeric RNAs in the same series employed slightly different target regions so that the appropriate bulge size could be created between the intergenic DNA stem and the RNA/DNA duplex (Figure 1A,C; Supplementary File S1). We kept the same bulge length on both sides of intergenic DNA stem, that is, if 2 nt was on the TMPRSS2 side, then 2 nt was also on the ERG side. All the designed chimeric RNAs contained a 75-nt targeting ERG gene and 52-nt targeting TMPRSS2 gene, so that they matched the length of the most potent antisense-5 chimeric RNA [6]. In all, thirty chimeric RNAs (3 locations × 10 different bulge sizes = 30 constructs) were designed and tested in LNCaP cells for their efficiency in inducing TMPRSS2-ERG gene fusion (Supplementary File S2).

**Figure 2:**
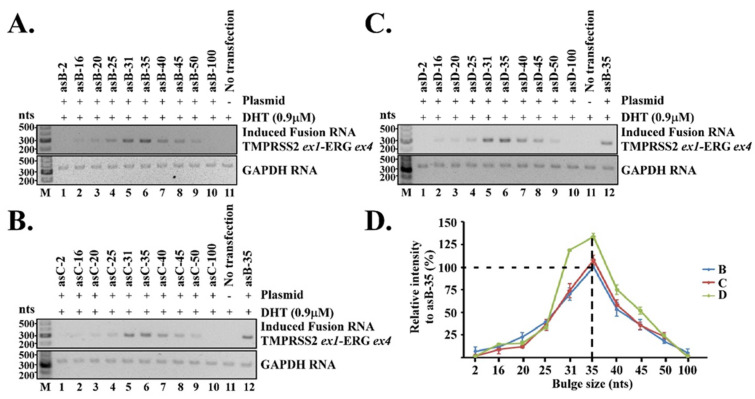
The bulge size regulated the efficiency of RNA-mediated gene fusion. A set of ten different antisense chimeric RNAs were designed to create different bulge sizes when annealed to each targeted location described in Figure 1. LNCaP cells were transfected with designed chimeric RNAs and treated with 900 nM of DHT for three days. RT-PCR was then performed to detect the level of induced TMPRSS2-ERG fusion RNA. GAPDH RNA was used as loading control. No transfection was used as the negative control for RT-PCR reactions. (**A**) RT-PCR results of induced TMPRSS2-ERG transcripts by chimeric RNAs designed to target location B. (**B**) RT-PCR results by chimeric RNAs designed to target location C. (**C**) RT-PCR results by chimeric RNAs designed to target location D. (**D**) All experiments were repeated independently thrice starting from cell transfection to RT-PCR and quantifications. Quantitation was done using ImageJ software. The average band intensities from three independent experiments were plotted as a line graph against the bulge size. Error bars represent standard deviations. The dashed line marks the most effective bulge size. The induced TMPRSS2-ERG fusion RNA level by antisense chimeric RNA ‘asB-35′ was used as the relative 100%.

We transiently expressed the chimeric RNAs in LNCaP cells by transfection, then treated the cells with DHT for three days. If the expression of the chimeric RNA led to a TMPRSS2-ERG gene fusion, it was expected that the endogenous full-length fusion RNAs would be transcribed from the newly induced TMPRSS2-ERG fusion gene. As we have pointed out in our previous study [6], the induced TMPRSS2-ERG fusion RNAs, which contain only annotated exon sequences, cannot arise from the sequence of the expression plasmids. This is because the chimeric RNA sequences encoded in the plasmids are designed to target the introns (Figure 1B) and contain no exon sequence. Second, the precise annotated splice junctions that join the exons as found in induced fusion transcripts (including the RNA junction that joins TMPRSS2 exon-1 to ERG exon-4) strongly indicate that they are generated and processed through cellular splicing mechanisms; therefore, the induced fusion transcript is not the result of RT-PCR artifacts produced by template switching. Specific nested RT-PCR primers were used to amplify the induced TMPRSS2-ERG fusion RNAs, and the levels of induced TMPRSS2-ERG fusion RNAs were then quantified. All experiments were repeated independently thrice starting from cell transfection to RT-PCR and quantifications (see Figure 2 and Supplementary Figure S1).

As shown in Figure 2A, when targeting location B, the chimeric RNA designed to create a bulge size of 35 nt (named ‘asB-35’) induced the maximum level of TMPRSS2-ERG fusion transcript. The intensity of induced TMPRSS2-ERG fusion transcript gradually tapered off when the bulge size was decreased or increased, and was nearly undetectable when the bulge size was reduced to 2 nt or increased to 100 nt. A similar pattern was also observed when targeting location C (Figure 2B) or location D (Figure 2C). Figure 2A–C are data obtained from a single experiment. We then averaged the induced TMPRSS2-ERG band intensities from all three independent experiments (see Supplementary Figure S1), and plotted them against the bulge size. As shown in Figure 2D, the overall pattern was nearly identical whether the chimeric RNAs were targeting locations B, C, or D. That is, a bulge size of 35 nt consistently induced the maximum level of TMPRSS2-ERG regardless of the target locations (therefore the target sequences) within TMPRSS2 and ERG genes. This observation was not due to the varied stability of RNA/DNA duplex that was used to create bulge, as the melting temperature (Tm) for all the chimeric RNAs in the same series were similar (see Supplementary Figure S2). Yet it was the chimeric RNA forming a 35-nt bulge in each series that induced the maximum level of TMPRSS2-ERG fusion RNA. Nor was the observation due to a specific bulge sequence, as each location created a completely different bulge sequence; yet it was the 35-nt bulge regardless of its sequence that consistently induced the maximum level of TMPRSS2-ERG fusion RNA in all locations. Together, the results suggest that the bulge size in the three-way junction strongly regulate the efficiency of RNA-mediated gene fusion, and a 35-nt bulge induced the maximum level of gene fusion.

### 3.2. Chimeric RNA Length Contributes to the Efficiency of RNA-Mediated Gene Fusion

**Figure 3:**
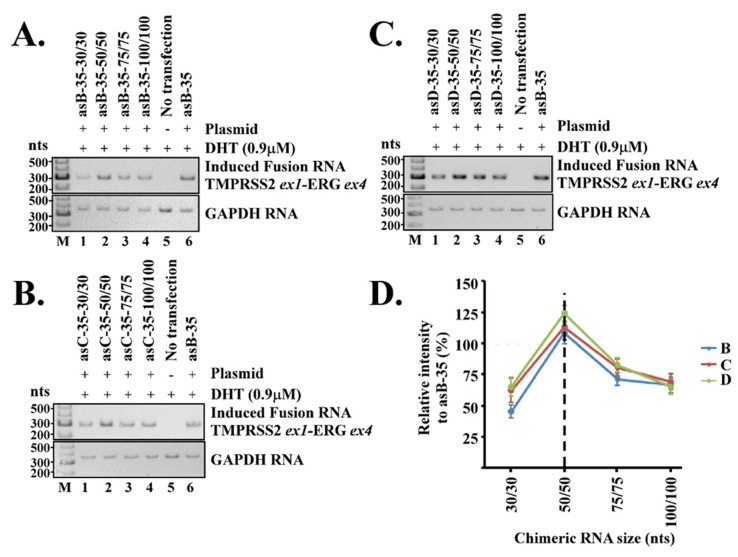
The length of chimeric RNA controls the efficiency of RNA-mediated gene fusion. A set of four different sized antisense chimeric RNAs was designed to target each location as described in Figure 1. Each set tested four different RNA lengths: 30/30, 50/50, 75/75, and 100/100 nt. The bulge size was fixed at 35 nt. (**A**) RT-PCR results of induced TMPRSS2-ERG transcripts by chimeric RNAs designed to target location B. (**B**) RT-PCR results by chimeric RNAs designed to target location C. (**C**) RT-PCR results by chimeric RNAs designed to target location D. (**D**) All experiments were repeated independently thrice starting from cell transfection to RT-PCR and quantifications. The average band intensities from three independent experiments were plotted as line graph against RNA length. Error bars represent standard deviations. The dashed line marks the most effective RNA length. The induced TMPRSS2-ERG fusion RNA level by antisense chimeric RNA ‘asB-35’ was used as the relative 100%.

After the effective bulge size was determined, we then set out to determine the lengths of chimeric RNA which contributed directly to the stability of the transient RNA/DNA duplex required for gene fusion induction. To determine the optimal RNA length, we designed three series of antisense chimeric RNAs with each series targeting locations B, C, and D. Each series tested four different RNA lengths: 30/30, 50/50, 75/75, and 100/100 nt. For example, a chimeric RNA with a length of 30/30 nt contains 30 nt complementary to ERG gene sequence followed by 30 nt complementary to TMPRSS2 gene sequence. To create different chimeric RNA lengths targeting each specific location, our designs maintained the same intergenic DNA stem, fixed the bulge size at 35 nt, and then employed progressively longer target sequences until the appropriate RNA lengths are reached (see Figure 1A,C for examples). Because locations B, C, and D were in completely different places in the introns of TMPRSS2 and ERG genes (Figure 1B), the chimeric RNAs targeting them also had completely different sequences. Therefore, these locations represented three independent experimental examples for which the optimal chimeric RNA length could be probed independently. In all, twelve chimeric RNAs (3 locations × 4 different lengths = 12 constructs) were designed and tested in LNCaP cells for their efficiency in inducing TMPRSS2-ERG fusion RNA. All experiments were repeated independently thrice starting from cell transfection to RT-PCR and quantifications (see Figure 3 and Supplementary Figure S3).

As shown in Figure 3A, when targeting location B, the chimeric RNA having a length of 50/50-nt (named ‘asB-35-50/50’) induced the highest level of TMPRSS2-ERG fusion transcript. The intensity of induced TMPRSS2-ERG fusion transcript gradually tapered off when the length was reduced to 30/30 nt or increased to 100/100 nt. Similarly, a chimeric RNA length of 50/50 nt also induced the highest level of TMPRSS2-ERG fusion transcript at location C (Figure 3B) and location D (Figure 3C). Figure 3A–C are data obtained from a single experiment. We then averaged the induced TMPRSS2-ERG band intensities from all three independent experiments (see Supplementary Figure S3), and plotted them against the RNA length. As shown in Figure 3D, the overall induction pattern remained similar whether the chimeric RNAs were targeting location B, C, or D. That is, a length of 50/50 nt seemed to induce the maximum level of TMPRSS2-ERG within the same series, regardless of the target locations within TMPRSS2 and ERG genes. This observation was not due to the specific target sequence, as each location had a completely different target sequence; yet it was the length of 50/50 nt that consistently induced the maximum level of TMPRSS2-ERG fusion RNA in all locations. Together, the results suggest that, while a broad range of chimeric RNA lengths from 30/30nt to 100/100 nt is capable of inducing gene fusion, an RNA length of 50/50 nt gives the maximum efficiency.

## 4. Discussion

Previously we reported an unappreciated RNA-driven mechanism in which the expression of a designer chimeric RNA induce specified gene fusions in mammalian cells [6,7]. The process as specified by the sequence of chimeric RNA involved, and over-expression of RNase-H, which degraded the RNA in an RNA/DNA duplex and significantly reduced the efficiency of RNA-induced gene fusion [6]. Furthermore, chimeric RNAs targeting genomic regions that can form stable intergenic DNA stems led to efficient gene fusion induction. In contrast, targeting regions having lower intergenic DNA stem stabilities resulted in no gene fusion induction [6]. These results suggest that the RNA/DNA duplex formed by chimeric RNA and its target genes may not be sufficiently stable, and additional elements such as the intergenic DNA stems are required to further stabilize the RNA/DNA duplex. This led to a working model where the chimeric RNA sequence invade the target genes to stabilize a higher-order structural motif resembling a three-way junction, which consists of the RNA/DNA duplex and the intergenic DNA stem (Figure 1A). Resolving such an structural motif by DNA break/repair mechanisms yields the final gene fusion through recombination in regions prone to DNA breaks.

Three-way junctions have long been proposed to play important roles in many biological mechanisms by stabilizing nucleic acid interactions [20,21,22]. In our model, a three-way junction keeps the two genomic loci in close proximity and further stabilizes the transient RNA/DNA duplex within. Our current report highlights the effects of two essential elements in the proposed three-way junction for RNA-mediated gene fusion: (1) the unpaired bulges linking between the RNA/DNA duplex and the intergenic DNA stem and (2) the chimeric RNA length for forming the RNA/DNA duplex. Our results indicate that both play important roles in regulating the efficiency of RNA-mediated gene fusion. The optimal length of an unpaired bulge, as determined empirically, is about 35 nt, while the optimal chimeric RNA length is about 50 nt for targeting. These parameters appear to consistently induce maximum level of TMPRSS2-ERG fusion RNA regardless of the target locations within TMPRSS2 and ERG genes.

Naturally occurring RNA molecules such as ribosomal RNAs contain numerous three-way junctions, most of which include stretches of unpaired nucleotides called “bulges” at the branch point [16,17]. These bulges provide flexible hinges, and are known to facilitate the coaxial stacking of the flanking stems in higher-order structures, which increases the stability of three-way junctions [18,19]. Studies of the folding of single RNA molecules found that bulges in three-way junctions vary frequently from one unpaired nucleotide up to several nucleotides [19,23]. However, the number of unpaired nucleotides in the bulge may depend on the type of stems and structures involved. In contrast to single RNA folding, our proposed three-way junction involves three molecules—two genomic DNA loci (such as TMPRSS2 and ERG) and an RNA. The bulge size required to stabilize such a large three-way junction complex might be different from that found in a single RNA molecule. Our results indicate that a bulge of 35 nt consistently induced the maximum level of TMPRSS2-ERG regardless of the target locations within TMPRSS2 and ERG genes (Figure 2D). Yet, gene fusion efficiency quickly diminished when the bulge size deviated from 35 nt, and was nearly undetectable when the bulge size was reduced to 2 nt or increased to 100 nt. The reasons for 35 nt being the optimal bulge size are not yet understood. Nonetheless, it is evident that the bulge size strongly regulates the efficiency of RNA-mediated gene fusion.

The lengths of chimeric RNA contribute directly to the stability of the transient RNA/DNA duplex. Our results show that chimeric RNA with a length of 50/50 nt is optimal in inducing TMPRSS2-ERG regardless of the target locations within TMPRSS2 and ERG genes. The intensity of induced the TMPRSS2-ERG fusion transcript gradually tapered off when the length was reduced to 30/30 nt or increased to 100/100 nt. This raises the question of why longer RNAs such as 100/100 nt are less efficient for RNA-mediated gene fusion as they should increase the stability of RNA/DNA duplex. Past studies of microarray technologies indicated that hybridization of DNA oligos to rRNA, which also forms an RNA/DNA duplex, is mainly affected by the secondary structures of the RNA molecules. The presence of secondary structures in RNA can reduce the binding of a DNA oligo by a factor of 10^5^ to 10^6^ [24,25]. In addition, when oligo sizes were reduced from 1480 nt to 45 nt, the hybridization efficiency increased several-fold [25]. Therefore, longer nucleic acids may create additional barriers for hybridization due to the increased probability of unwanted secondary structures.

A second intriguing question is whether a short chimeric RNA of 30/30-nt is sufficient to specify two parental genes for gene fusion. The well-studied guide RNA used in CRISPR technology only has a 20-nt target recognition sequence [26,27]. Yet, such a length is sufficient to specify a location within the genome albeit with known off-target issues. A chimeric RNA of 30/30 nt has a target recognition sequence substantially longer than 20 nt, which should be sufficient to specify two parental genes for gene fusion and a chimeric RNA of 50/50 nt should be more than sufficient to specify two parental genes. Consistent with this are the empirical results that chimeric RNAs designed to target TMPRSS2 and ERG genes with lengths of either 30/30 nt or 50/50 nt were capable of inducing the intended TMPRSS2-ERG gene fusion (Figure 3).

In summary, by focusing on three independent targeting locations, we showed that both the unpaired bulge sizes and the chimeric RNA lengths play important roles in RNA-mediated gene fusion. The optimal length of an unpaired bulge is about 35 nt, while the optimal chimeric RNA length is about 50 nt for targeting. These observations are consistent regardless of the target locations within TMPRSS2 and ERG genes, with each location involving different targeted sequences and different bulge sequences. These empirically determined parameters for RNA-mediated gene fusion are important, as they provide valuable insight for formulating bioinformatics guidelines to search for cellular chimeric-like RNAs that may initiate oncogenic fusion genes. Secondly, these parameters provide the design principles for developing new technology for manipulating the mammalian genome through large-distance DNA rearrangements (as opposed to the local base-editing offered by CRISPR technology). Lastly, this knowledge could facilitate the development of therapeutic strategies to inhibit the formation of RNA/DNA duplexes and three-way junctions, therefore preventing the formation of cancer fusion genes and future tumors.

## Data Availability

Data can be found online as supplementary file.

## Supplementary Materials

The following supporting information can be downloaded at: https://www.mdpi.com/article/10.3390/cells11061002/s1, Figure S1: The bulge size regulates the efficiency of RNA-mediated gene fusion; Figure S2: The melting temperature (Tm) of chimeric RNA designed to create different bulge sizes; Figure S3: The length of chimeric RNA controls the efficiency of RNA-mediated gene fusion; Figure S4: The melting temperature (Tm) of chimeric RNA with different target lengths; File S1: Genomic sequences targeted by designed chimeric RNA with different bulge sizes; File S2: Chimeric RNA sequences.
