# Supplementary material for: Chimeric RNA Design Principles for RNA-Mediated Gene Fusion
Source: Cells. 2022 Mar 16;11(6):1002. doi: 10.3390/cells11061002 (PMC8947500; doi:10.3390/cells11061002)
Supplement: Supplementary file 1 [file cells-11-01002-s001.zip › cells-1619768-supplementary.pdf]

cells MDPI

Supplementary Information

Chimeric RNA design principles for RNA-mediated gene fusion

Sachin Kumar Gupta and Laising Yen*

[1.] Department of Pathology & Immunology, Baylor College of Medicine, Houston, TX 77030, USA; sachinkumar.gupta@bcm.edu
[2] Department of Molecular & Cellular Biology, Baylor College of Medicine, Houston, TX 77030, USA
[3] Dan L. Duncan Cancer Center, Baylor College of Medicine, Houston, TX 77030, USA
* Correspondence: author: yen@bcm.edu; Tel.: +1-713-798-1180

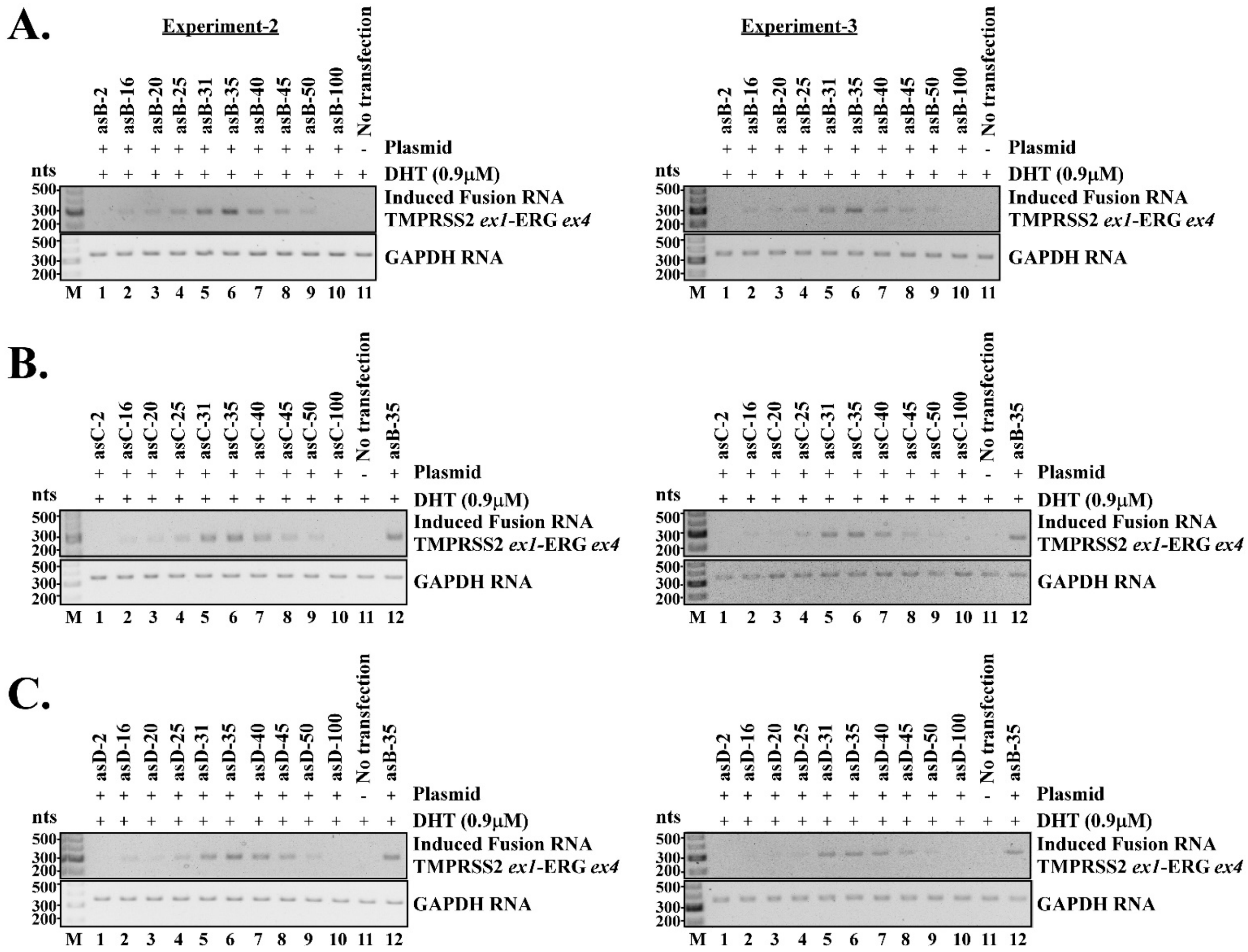

Supplementary Figure S1. The bulge size regulates the efficiency of RNA-mediated gene fusion.

The RT-PCR gels shown in Figure 1 in the main text were obtained from a single experiment. Here we show the RT-PCR gels from additional two independent experiments. **A.** RT-PCT results of induced TMPRSS2-ERG transcripts by chimeric RNAs designed to target location B. **B.** RT-PCT results by chimeric RNAs designed to target location C. **C.** RT-PCT results by chimeric RNAs designed to target location D. GAPDH RNA was used as loading control. No transfection was used as the negative control for RT-PCR reactions. Left panel: Experiment-2, Right panel: Experiment-3.

A.	Tm (ºC) for targeting TMPRSS2 genomic region	Tm (ºC) for targeting ERG genomic region
asB-2	85	75
asB-16	86	74
asB-20	87	73
asB-25	88	73
asB-31	88	74
asB-35	86	75
asB-40	87	76
asB-45	87	76
asB-50	87	76
asB-100	79	75

B.	Tm (ºC) for targeting TMPRSS2 genomic region	Tm (ºC) for targeting ERG genomic region
asC-2	81	73
asC-16	83	75
asC-20	84	74
asC-25	84	73
asC-31	83	73
asC-35	83	74
asC-40	84	75
asC-45	82	73
asC-50	80	74
asC-100	78	75

C.	Tm (ºC) for targeting TMPRSS2 genomic region	Tm (ºC) for targeting ERG genomic region
asD-2	88	72
asD-16	86	72
asD-20	87	74
asD-25	86	74
asD-31	85	73
asD-35	85	73
asD-40	83	72
asD-45	82	73
asD-50	84	72
asD-100	86	75

Supplementary Figure S2. The melting temperature (Tm) of chimeric RNA designed to create different bulge sizes. Tm was calculated using formula: Tm= 64.9 +41*(nG+nC-16.4)/(nA+nT+nG+nC) where "n" stands for total number of particular nucleotide. The Tm calculation can be found at this link: http://insilico.ehu.es/tm.php?formula=basic.

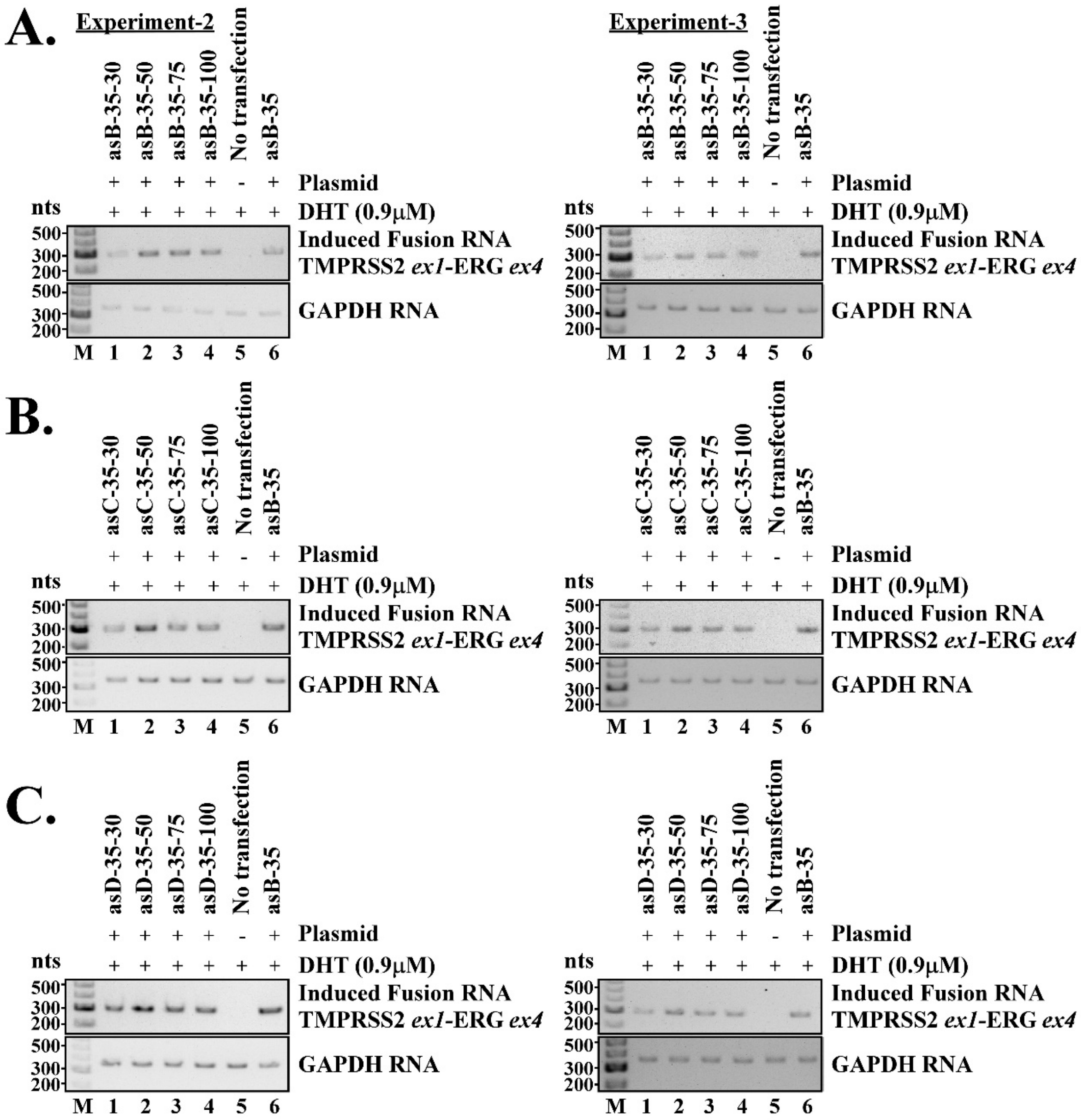

Supplementary Figure S3. The length of chimeric RNA controls the efficiency of RNA-mediated gene fusion.

The RT-PCR gels shown in Figure 2 in the main text were obtained from a single experiment. Here we show the RT-PCR gels from additional two independent experiments. **A.** RT-PCT results of induced TMPRSS2-ERG transcripts by chimeric RNAs designed to target location B. **B.** RT-PCT results by chimeric RNAs designed to target location C. **C.** RT-PCT results by chimeric RNAs designed to target location D. GAPDH RNA was used as loading control. No transfection was used as the negative control for RT-PCR reactions. Left panel: Experiment-2, Right panel: Experiment-3.

A.	Tm (°C) for targeting TMPRSS2 genomic region	Tm (°C) for targeting ERG genomic region
asB-35-30/30	77	65
asB-35-50/50	86	69
asB-35-75/75	88	75
asB-35-100/100	91	77

B.	Tm (°C) for targeting TMPRSS2 genomic region	Tm (°C) for targeting ERG genomic region
asC-35-30/30	79	63
asC-35-50/50	83	72
asC-35-75/75	85	74
asC-35-100/100	86	76

C.	Tm (°C) for targeting TMPRSS2 genomic region	Tm (°C) for targeting ERG genomic region
asD-35-30/30	75	61
asD-35-50/50	84	69
asD-35-75/75	88	73
asD-35-100/100	91	75

Supplementary Figure S4. The melting temperature (Tm) of chimeric RNA with different target lengths. Tm was calculated using formula: Tm= 64.9 +41*(nG+nC-16.4)/(nA+nT+nG+nC) where "n" stands for total number of particular nucleotide. The Tm calculation can be found at this link: http://insilico.ehu.es/tm.php?formula=basic.

Supplementary file S1: Genomic sequences targeted by designed chimeric RNA with different bulge sizes

Location B:

B-2

TMPRSS2

Tgagattaaagcgagagccagggcgggccgggccgagtaggcgcgagctaagcaggaggcggaggcggaggcggagggcgaggggcg
gggagcgccgcctggagcgcggcaggtgagcggcgccggtaccagggtcccggctcggggtccgggctggggaggg

: targeted TMPRSS2 sequence, : bulge, : stem

ERG

Ccctccccagctcacgaggggtggccccacttctatgcttttccttgcagtttctccatctggaaatgctccccaccattttgattt
acccagtacaccccattcttcaaggctcaggccagtgcacaccagttcctttaatccctccttccttcttctgtcatccagtcgaca
cacttagcatga

: targeted ERG sequence, : bulge, : stem

B-16

TMPRSS2

Tgagattaaagcgagagccagggcgggccgggccgagtaggcgcgagctaagcaggaggcggaggcggaggcggagggcgaggggcg
gggagcgccgcctggagcgcggcaggtgagcggcgccggtaccagggtcccggctcggggtccgggctggggaggg

: targeted TMPRSS2 sequence, : bulge, : stem

ERG

Ccctccccagctcacgaggggtggccccacttctatgcttttccttgcagtttctccatctggaaatgctccccaccattttgattt
acccagtacaccccattcttcaaggctcaggccagtgcacaccagttcctttaatccctccttccttcttctgtcatccagtcgaca
cacttagcatga

: targeted ERG sequence, : bulge, : stem

B-20

TMPRSS2

Tgagattaaagcgagagccagggcgggccgggccgagtaggcgcgagctaagcaggaggcggaggcggaggcggagggcgaggggcg
gggagcgccgcctggagcgcggcaggtgagcggcgccggtaccagggtcccggctcggggtccgggctggggaggg

: targeted TMPRSS2 sequence, : bulge, : stem

ERG

Ccctccccagctcacgaggggtggccccacttctatgcttttccttgcagtttctccatctggaaatgctccccaccattttgatttacccagtacaccccattcttcaaggctcaggccagtgcacaccagttcctttaatccctccttccttcttctgtcatccagtcgacacacttagcatga

: targeted ERG sequence, : bulge, : stem

B-25

TMPRSS2

Tgagattaaagcgagagccagggcgggccgggccgagtaggcgcgagctaagcaggaggcggaggcggaggcggagggcgaggggcggggagcgccgcctggagcgcggcaggtgagcggcgccggtaccagggtcccggctcggggtccgggctggggaggg

: targeted TMPRSS2 sequence, : bulge, : stem

ERG

Ccctccccagctcacgaggggtggccccacttctatgcttttccttgcagtttctccatctggaaatgctccccaccattttgatttacccagtacaccccattcttcaaggctcaggccagtgcacaccagttcctttaatccctccttccttcttctgtcatccagtcgacacacttagcatga

: targeted ERG sequence, : bulge, : stem

B-31

TMPRSS2

Tgagattaaagcgagagccagggcgggccgggccgagtaggcgcgagctaagcaggaggcggaggcggaggcggagggcgaggggcggggagcgccgcctggagcgcggcaggtgagcggcgccggtaccagggtcccggctcggggtccgggctggggaggg

: targeted TMPRSS2 sequence, : bulge, : stem

ERG

Ccctccccagctcacgaggggtggccccacttctatgcttttccttgcagtttctccatctggaaatgctccccaccattttgatttacccagtacaccccattcttcaaggctcaggccagtgcacaccagttcctttaatccctccttccttcttctgtcatccagtcgacacacttagcatga

: targeted ERG sequence, : bulge, : stem

B-35

TMPRSS2

Tgagattaaagcgagagccagggcgggccgggccgagtaggcgcgagctaagcaggaggcggaggcggaggcggagggcgaggggcg
gggagcgccgcctggagcgcggcaggtgagcggcgccggtaccagggtcccggctcggggtccgggctggggaggg

: targeted TMPRSS2 sequence, : bulge, : stem

ERG

Ccctccccagctcacgaggggtggccccacttctatgcttttccttgcagtttctccatctggaaatgctccccaccattttgattt
acccagtacaccccattcttcaaggctcaggccagtgcacaccagttcctttaatccctccttccttcttctgtcatccagtcgaca
cacttagcatga

: targeted ERG sequence, : bulge, : stem

B-40

TMPRSS2

Tgagattaaagcgagagccagggcgggccgggccgagtaggcgcgagctaagcaggaggcggaggcggaggcggagggcgaggggcg
gggagcgccgcctggagcgcggcaggtgagcggcgccggtaccagggtcccggctcggggtccgggctggggaggg

: targeted TMPRSS2 sequence, : bulge, : stem

ERG

Ccctccccagctcacgaggggtggccccacttctatgcttttccttgcagtttctccatctggaaatgctccccaccattttgattt
acccagtacaccccattcttcaaggctcaggccagtgcacaccagttcctttaatccctccttccttcttctgtcatccagtcgaca
cacttagcatga

: targeted ERG sequence, : bulge, : stem

B-45

TMPRSS2

Tgagattaaagcgagagccagggcgggccgggccgagtaggcgcgagctaagcaggaggcggaggcggaggcggagggcgaggggcg
gggagcgccgcctggagcgcggcaggtgagcggcgccggtaccagggtcccggctcggggtccgggctggggaggg

: targeted TMPRSS2 sequence, : bulge, : stem

ERG

Ccctccccagctcacgaggggtggccccacttctatgcttttccttgcagtttctccatctggaaatgctccccaccattttgattt acccagtacaccccattcttcaaggctcaggccagtgcacaccagttcctttaatccctccttccttcttctgtcatccagtcgaca cacttagcatga

: targeted ERG sequence, : bulge, : stem

B-50

TMPRSS2

Tgagattaaagcgagagccagggcgggccgggccgagtaggcgcgagctaagcaggaggcggaggcggaggcggagggcgaggggcg gggagcgccgcctggagcgcggcaggtgagcggcgccggtaccagggtcccggctcggggtccgggctggggaggg

: targeted TMPRSS2 sequence, : bulge, : stem

ERG

Ccctccccagctcacgaggggtggccccacttctatgcttttccttgcagtttctccatctggaaatgctccccaccattttgattt acccagtacaccccattcttcaaggctcaggccagtgcacaccagttcctttaatccctccttccttcttctgtcatccagtcgaca cacttagcatga

: targeted ERG sequence, : bulge, : stem

B-100

TMPRSS2

Tgagattaaagcgagagccagggcgggccgggccgagtaggcgcgagctaagcaggaggcggaggcggaggcggagggcgaggggcg gggagcgccgcctggagcgcggcaggtgagcggcgccggtaccagggtcccggctcggggtccgggctggggaggg

: targeted TMPRSS2 sequence, : bulge, : stem

ERG

Ccctccccagctcacgaggggtggccccacttctatgcttttccttgcagtttctccatctggaaatgctccccaccattttgattt acccagtacaccccattcttcaaggctcaggccagtgcacaccagttcctttaatccctccttccttcttctgtcatccagtcgaca cacttagcatga

: targeted ERG sequence, : bulge, : stem

Location C:

C-2

TMPRSS2

Catcgcgaggttggggcaggtggtcctgcgagtccctagccagttggtggaagagagtcccccggggtccccaaagctggctcctag tccgcctgccctccacggcccccgcctgggagcaccggtgcgcctttctctttggggaggaggactgggagtgctg

: targeted TMPRSS2 sequence, : bulge, : stem

ERG

Cagcactcccagagtcataaaaatcaagtctcactggcaaccaacttgttagtgcatgaaaagtgttaccaggacggggtctaccca gtgctcatctatgtcataggaaaagcatgagctcagcgtataacattagcaaagggctctgatgacctggctacttagtcccctttc catgcagcaacca

: targeted ERG sequence, : bulge, : stem

C-16

TMPRSS2

Catcgcgaggttggggcaggtggtcctgcgagtccctagccagttggtggaagagagtcccccggggtccccaaagctggctcctag tccgcctgccctccacggcccccgcctgggagcaccggtgcgcctttctctttggggaggaggactgggagtgctg

: targeted TMPRSS2 sequence, : bulge, : stem

ERG

Cagcactcccagagtcataaaaatcaagtctcactggcaaccaacttgttagtgcatgaaaagtgttaccaggacggggtctaccca gtgctcatctatgtcataggaaaagcatgagctcagcgtataacattagcaaagggctctgatgacctggctacttagtcccctttc catgcagcaacca

: targeted ERG sequence, : bulge, : stem

C-20

TMPRSS2

Catcgcgaggttggggcaggtggtcctgcgagtccctagccagttggtggaagagagtcccccggggtccccaaagctggctcctag tccgcctgccctccacggcccccgcctgggagcaccggtgcgcctttctctttggggaggaggactgggagtgctg

: targeted TMPRSS2 sequence, : bulge, : stem

ERG
Cagcactcccagagtcataaaaatcaagtctcactggcaaccaacttgttagtgcatgaaaagtgttaccaggacggggtctaccca
gtgctcatctatgtcataggaaaagcatgagctcagcgtataacattagcaaagggctctgatgacctggctacttagtcccctttc
catgcagcaacca

: targeted ERG sequence, : bulge, : stem

C-25

TMPRSS2
Catcgcgaggttggggcaggtggtcctgcgagtccctagccagttggtggaagagagtcccccggggtccccaaagctggctcctag
tccgcctgccctccacggcccccgcctgggagcaccggtgcgcctttctctttggggaggaggactgggagtgctg

: targeted TMPRSS2 sequence, : bulge, : stem

ERG
Cagcactcccagagtcataaaaatcaagtctcactggcaaccaacttgttagtgcatgaaaagtgttaccaggacggggtctaccca
gtgctcatctatgtcataggaaaagcatgagctcagcgtataacattagcaaagggctctgatgacctggctacttagtcccctttc
catgcagcaacca

: targeted ERG sequence, : bulge, : stem

C-31

TMPRSS2
Catcgcgaggttggggcaggtggtcctgcgagtccctagccagttggtggaagagagtcccccggggtccccaaagctggctcctag
tccgcctgccctccacggcccccgcctgggagcaccggtgcgcctttctctttggggaggaggactgggagtgctg

: targeted TMPRSS2 sequence, : bulge, : stem

ERG
Cagcactcccagagtcataaaaatcaagtctcactggcaaccaacttgttagtgcatgaaaagtgttaccaggacggggtctaccca
gtgctcatctatgtcataggaaaagcatgagctcagcgtataacattagcaaagggctctgatgacctggctacttagtcccctttc
catgcagcaacca

: targeted ERG sequence, : bulge, : stem

C-35

TMPRSS2

Catcgcgaggttggggcaggtggtcctgcgagtccctagccagttggtggaagagagtcccccggggtccccaaagctggctcctag
tccgcctgccctccacggcccccgcctggg agcaccggtgcgcctttctctttggggaggaggactgggagtgctg

: targeted TMPRSS2 sequence, : bulge, : stem

ERG

Cagcactcccagagtcataaaatcaagtctcactggcaaccaacttgttagtgcatgaaaagtgttaccaggacggggtctaccca
gtgctcatctatgtcataggaaaagcatgagctcagcgtataacattagcaaagggctctgatgacctggctacttagtcccctttc
catgcagcaacca

: targeted ERG sequence, : bulge, : stem

C-40

TMPRSS2

Catcgcgaggttggggcaggtggtcctgcgagtccctagccagttggtggaagagagtcccccggggtccccaaagctggctcctag
tccgcctgccctccacggcccccgcctgggagcaccggtgcgcctttctctttggggaggaggactgggagtgctg

: targeted TMPRSS2 sequence, : bulge, : stem

ERG

Cagcactcccagagtcataaaatcaagtctcactggcaaccaacttgttagtgcatgaaaagtgttaccaggacggggtctaccca
gtgctcatctatgtcataggaaaagcatgagctcagcgtataacattagcaaagggctctgatgacctggctacttagtcccctttc
catgcagcaacca

: targeted ERG sequence, : bulge, : stem

C-45

TMPRSS2

Catcgcgaggttggggcaggtggtcctgcgagtccctagccagttggtggaagagagtcccccggggtccccaaagctggctcctag
tccgcctgccctccacggcccccgcctgggagcaccggtgcgcctttctctttggggaggaggactgggagtgctg

: targeted TMPRSS2 sequence, : bulge, : stem

ERG

Cagcactcccagagtcataaaaatcaagtctcactggcaaccaacttgttagtgcatgaaaagtgttaccaggacggggtctaccca
gtgctcatctatgtcataggaaaagcatgagctcagcgtataacattagcaaagggctctgatgacctggctacttagtcccctttc
catgcagcaacca

: targeted ERG sequence, : bulge, : stem

C-50

TMPRSS2

Catcgcgaggttggggcaggtggtcctgcgagtccctagccagttggtggaagagagtcccccggggtccccaaagctggctcctag
tccgcctgccctccacggcccccgcctgggagcaccggtgcgcctttctctttggggaggaggactgggagtgctg

: targeted TMPRSS2 sequence, : bulge, : stem

ERG

Cagcactcccagagtcataaaaatcaagtctcactggcaaccaacttgttagtgcatgaaaagtgttaccaggacggggtctaccca
gtgctcatctatgtcataggaaaagcatgagctcagcgtataacattagcaaagggctctgatgacctggctacttagtcccctttc
catgcagcaacca

: targeted ERG sequence, : bulge, : stem

C-100

TMPRSS2

Catcgcgaggttggggcaggtggtcctgcgagtccctagccagttggtggaagagagtcccccggggtccccaaagctggctcctag
tccgcctgccctccacggcccccgcctgggagcaccggtgcgcctttctctttggggaggaggactgggagtgctg

: targeted TMPRSS2 sequence, : bulge, : stem

ERG

Cagcactcccagagtcataaaaatcaagtctcactggcaaccaacttgttagtgcatgaaaagtgttaccaggacggggtctaccca
gtgctcatctatgtcataggaaaagcatgagctcagcgtataacattagcaaagggctctgatgacctggctacttagtcccctttc
catgcagcaacca

: targeted ERG sequence, : bulge, : stem

Location D:

D-2

TMPRSS2

Gctggggaggggaacctgggcgcctgggacccgccgatgccccctgccccgcccggaggtgaaagcgggtgtgaggagcgcggcgcg gcaggtgagtgcgcccgagggtcgagcgctggggccagccgggcagggcgcctcccggggtgctgggagagtgctggg

: targeted TMPRSS2 sequence, : bulge, : stem

ERG

Cccagcactctccgtttaccatccagatcctttattccatcccagatccagggattctggacacttatcccttcatctttcttctgt ctcttccctctaccagcctctctctctctgtctctctctaggtgattccatctctcatggcaagagcaggcaccaaaacactccacc gtgtgacttttctt

: targeted ERG sequence, : bulge, : stem

D-16

TMPRSS2

Gctggggaggggaacctgggcgcctgggacccgccgatgccccctgccccgcccggaggtgaaagcgggtgtgaggagcgcggcgcg gcaggtgagtgcgcccgagggtcgagcgctggggccagccgggcagggcgcctcccggggtgctgggagagtgctggg

: targeted TMPRSS2 sequence, : bulge, : stem

ERG

Cccagcactctccgtttaccatccagatcctttattccatcccagatccagggattctggacacttatcccttcatctttcttctgt ctcttccctctaccagcctctctctctctgtctctctctaggtgattccatctctcatggcaagagcaggcaccaaaacactccacc gtgtgacttttctt

: targeted ERG sequence, : bulge, : stem

D-20

TMPRSS2

Gctggggaggggaacctgggcgcctgggacccgccgatgccccctgccccgcccggaggtgaaagcgggtgtgaggagcgcggcgcg
gcaggtgagtgcgcccgagggtcgagcgctggggccagccgggcagggcgcctcccggggtgctgggagagtgctggg

: targeted TMPRSS2 sequence, : bulge, : stem

ERG
Cccagcactctccgtttaccatccagatcctttattccatcccagatccagggattctggacacttatcccttcatctttcttctgt
ctcttccctctaccagcctctctctctgtctctctctaggtgattccatctctcatggcaagagcaggcaccaaaacactccacc
gtgtgacttttctt

: targeted ERG sequence, : bulge, : stem

D-25

TMPRSS2
Gctggggaggggaacctgggcgcctgggacccgccgatgccccctgccccgcccggaggtgaaagcgggtgtgaggagcgcggcgcg
gcaggtgagtgcgcccgagggtcgagcgctggggccagccgggcagggcgcctcccggggtgctgggagagtgctggg

: targeted TMPRSS2 sequence, : bulge, : stem

ERG
Cccagcactctccgtttaccatccagatcctttattccatcccagatccagggattctggacacttatcccttcatctttcttctgt
ctcttccctctaccagcctctctctctgtctctctctaggtgattccatctctcatggcaagagcaggcaccaaaacactccacc
gtgtgacttttctt

: targeted ERG sequence, : bulge, : stem

D-31

TMPRSS2
Gctggggaggggaacctgggcgcctgggacccgccgatgccccctgccccgcccggaggtgaaagcgggtgtgaggagcgcggcgcg
gcaggtgagtgcgcccgagggtcgagcgctggggccagccgggcagggcgcctcccggggtgctgggagagtgctggg

: targeted TMPRSS2 sequence, : bulge, : stem

ERG

Cccagcactctccgtttaccatccagatcctttattccatcccagatccaggattctggacacttatcccttcatctttcttctgt
ctcttccctctaccagcctctctctctctgtctctctctaggtgattccatctctcatggcaagagcaggcaccaaaacactccacc
gtgtgacttttctt

: targeted ERG sequence, : bulge, : stem

D-35

TMPRSS2

Gctggggaggggaacctgggcgcctgggacccgccgatgcccccctgccccgcccggaggtgaaagcgggtgtgaggagcgcggcgcg
gcaggtgagtgcgcccgagggtcgagcgctggggccagccgggcagggcgcctcccggggtgctgggagagtgctggg

: targeted TMPRSS2 sequence, : bulge, : stem

ERG

Cccagcactctccgtttaccatccagatcctttattccatcccagatccaggattctggacacttatcccttcatctttcttctgt
ctcttccctctaccagcctctctctctctgtctctctctaggtgattccatctctcatggcaagagcaggcaccaaaacactccacc
gtgtgacttttctt

: targeted ERG sequence, : bulge, : stem

D-40

TMPRSS2

Gctggggaggggaacctgggcgcctgggacccgccgatgcccccctgccccgcccggaggtgaaagcgggtgtgaggagcgcggcgcg
gcaggtgagtgcgcccgagggtcgagcgctggggccagccgggcagggcgcctcccggggtgctgggagagtgctggg

: targeted TMPRSS2 sequence, : bulge, : stem

ERG

Cccagcactctccgtttaccatccagatcctttattccatcccagatccaggattctggacacttatcccttcatctttcttctgt
ctcttccctctaccagcctctctctctctgtctctctctaggtgattccatctctcatggcaagagcaggcaccaaaacactccacc
gtgtgacttttctt

: targeted ERG sequence, : bulge, : stem

D-45

TMPRSS2

Gctggggaggggaacctgggcgcctgggacccgccgatgccccctgccccgcccggaggtgaaagcgggtgtgaggagcgcggcgcg
gcaggtgagtgcgcccgagggtcgagcgctggggccagccgggcagggcgcctcccggggtgctgggagagtgctggg

: targeted TMPRSS2 sequence, : bulge, : stem

ERG

Cccagcactctccgtttaccatccagatcctttattccatcccagatccagggattctggacacttatcccttcatctttcttctgt
ctcttccctctaccagcctctctctctctgtctctctctaggtgattccatctctcatggcaagagcaggcaccaaaacactccacc
gtgtgacttttctt

: targeted ERG sequence, : bulge, : stem

D-50

TMPRSS2

Gctggggaggggaacctgggcgcctgggacccgccgatgccccctgccccgcccggaggtgaaagcgggtgtgaggagcgcggcgcg
gcaggtgagtgcgcccgagggtcgagcgctggggccagccgggcagggcgcctcccggggtgctgggagagtgctggg

: targeted TMPRSS2 sequence, : bulge, : stem

ERG

Cccagcactctccgtttaccatccagatcctttattccatcccagatccagggattctggacacttatcccttcatctttcttctgt
ctcttccctctaccagcctctctctctctgtctctctctaggtgattccatctctcatggcaagagcaggcaccaaaacactccacc
gtgtgacttttctt

: targeted ERG sequence, : bulge, : stem

D-100

TMPRSS2

Gctggggaggggaacctgggcgcctgggacccgccgatgccccctgccccgcccggaggtgaaagcgggtgtgaggagcgcggcgcg
gcaggtgagtgcgcccgagggtcgagcgctggggccagccgggcagggcgcctcccggggtgctgggagagtgctggg

: targeted TMPRSS2 sequence, : bulge, : stem

ERG

Cccagcactctccgtttaccatccagatcctttattccatcccagatccagggattctggacttatcccttcatctttcttctgt
ctcttccctctaccagcctctctctctctgtctctctctaggtgattccatctctcatggcaagagcaggcaccaaaacactccacc
gtgtgacttttctt

: targeted ERG sequence, : bulge, : stem

Supplementary file S2: Chimeric RNA sequences

asB-2

+1gtgctcgcttcggcagcacatatactaacattggaacgatcctgcagTAAATCAAAATGGTGGGGAGCATGTCCAGATGGAGAAA
CTGCAAGGAAAAGCATAGAAGTGGGGCCACCCCTCGTGGACCCCGAGCCGGGACCCTGGTACCGGCGCCGCTCACCTGCCGCGCTCC
AG

: *TMPRSS2*, : *ERG*, **+1**: Transcription start, ctgcag: PstI

asB-16

+1gtgctcgcttcggcagcacatatactaacattggaacgatcctgcagTGGGGTGTACTGGGTAAATCAAAATGGTGGGGAGCATT
TCCAGATGGAGAAACTGCAAGGAAAAGCATAGAAGTGGACCCTGGTACCGGCGCCGCTCACCTGCCGCGCTCCAGGCGGCGCTCCCC
GC

: *TMPRSS2*, : *ERG*, **+1**: Transcription start, ctgcag: PstI

asB-20

+1gtgctcgcttcggcagcacatatactaacattggaacgatcctgcag**AGAATGGGGTGTACTGGGTAAATCAAAATGGTGGGG**
AGCATTTCCAGATGGAGAAACTGCAAGGAAAAGCATAGACTGGTACCGGCGCCGCTCACCTGCCGCGCTCCAGGCGGCGCTCC
CCGCCCCT

: *TMPRSS2*, : *ERG*, **+1**: Transcription start, ctgcag: PstI

asB-25

+1gtgctcgcttcggcagcacatatactaacattggaacgatcctgcagCTTGAAGAATGGGGTGTACTGGGTAAATCAAAATGGTG
GGGAGCATTTCCAGATGGAGAAACTGCAAGGAAAAGCACCGGCGCCGCTCACCTGCCGCGCTCCAGGCGGCGCTCCCCGCCCCTCGC
CC

: *TMPRSS2*, : *ERG*, **+1**: Transcription start, ctgcag: PstI

asB-31

+1gtgctcgcttcggcagcacatatactaacattggaacgatcctgcag**CTGAGCCTTGAAGAATGGGGTGTACTGGGTAAATCA**
AAATGGTGGGGAGCATTTCCAGATGGAGAAACTGCAAGGGCCGCTCACCTGCCGCGCTCCAGGCGGCGCTCCCCGCCCCTCGC
CCTCCGCC

: *TMPRSS2*, : *ERG*, **+1**: Transcription start, ctgcag: PstI

asB-35

+1gtgctcgcttcggcagcacatatactaacattggaacgatc<u>ctgcag</u>**TGGCCTGAGCCTTGAAGAATGGGGTGTACTGGGTAA**
ATCAAAATGGTGGGGAGCATTTCCAGATGGAGAAACTGCCTCACCTGCCGCGCTCCAGGCGGCGCTCCCCGCCCCTCGCCCTC
CGCCTCCG

: *TMPRSS2*, : *ERG*, **+1**: Transcription start, ctgcag: PstI

asB-40

+1gtgctcgcttcggcagcacatatactaacattggaacgatc<u>ctgcag</u>TGCACTGGCCTGAGCCTTGAAGAATGGGGTGTACTGGG
TAAATCAAAATGGTGGGGAGCATTTCCAGATGGAGAACTGCCGCGCTCCAGGCGGCGCTCCCCGCCCCTCGCCCTCCGCCTCCGCCT
CC

: *TMPRSS2*, : *ERG*, **+1**: Transcription start, ctgcag: PstI

asB-45

+1gtgctcgcttcggcagcacatatactaacattggaacgatc<u>ctgcag</u>TGGTGTGCACTGGCCTGAGCCTTGAAGAATGGGGTGTA
CTGGGTAAATCAAAATGGTGGGGAGCATTTCCAGATGGCGCTCCAGGCGGCGCTCCCCGCCCCTCGCCCTCCGCCTCCGCCTCCGCC
TC

: *TMPRSS2*, : *ERG*, **+1**: Transcription start, ctgcag: PstI

asB-50

+1gtgctcgcttcggcagcacatatactaacattggaacgatc<u>ctgcag</u>GGAACTGGTGTGCACTGGCCTGAGCCTTGAAGAATGGG
GTGTACTGGGTAAATCAAAATGGTGGGGAGCATTTCCCCAGGCGGCGCTCCCCGCCCCTCGCCCTCCGCCTCCGCCTCCGCCTCCTG
CT

: *TMPRSS2*, : *ERG*, **+1**: Transcription start, ctgcag: PstI

asB-100

+1gtgctcgcttcggcagcacatatactaacattggaacgatcctgcagTCATGCTAAGTGTGTCGACTGGATGACAGAAGAAGGAA
GGAGGGATTAAAGGAACTGGTGTGCACTGGCCTGAGCCTTAGCTCGCGCCTACTCGGCCCGGCCCGCCCTGGCTCTCGCTTTAATCT
CA

: *TMPRSS2*, : *ERG*, **+1**: Transcription start, ctgcag: PstI

asC-2

+1gtgctcgcttcggcagcacatatactaacattggaacgatcctgcagACTGGGTAGACCCCGTCCTGGTAACACTTCTCATGCAC
TAACAAGTTGGTTGCCAGTGAGACTTGATTCTTATGACTCCTCCCCAAAGAGAAAAGGCGCACCGGTGCTCCCAGGCGGGGGCCGTG
GA

: *TMPRSS2*, : *ERG*, **+1**: Transcription start, ctgcag: PstI

asC-16

+1gtgctcgcttcggcagcacatatactaacattggaacgatcctgcag**TGACATAGATGAGCACTGGGTAGACCCCGTCCTGGT**
AACACTCTTCATGCACTAACAAGTTGGTTGCCAGTGAGAGAAAAGGCGCACCGGTGCTCCCAGGCGGGGGCCGTGGAGGGCAG
GCGGACTA

: *TMPRSS2*, : *ERG*, **+1**: Transcription start, ctgcag: PstI

asC-20

+1gtgctcgcttcggcagcacatatactaacattggaacgatcctgcag**CCTATGACATAGATGAGCACTGGGTAGACCCCGTCC**
TGGTAACACTCTTCATGCACTAACAAGTTGGTTGCCAGTAGGCGCACCGGTGCTCCCAGGCGGGGGCCGTGGAGGGCAGGCGG
ACTAGGAG

: *TMPRSS2*, : *ERG*, **+1**: Transcription start, ctgcag: PstI

asC-25

+1gtgctcgcttcggcagcacatatactaacattggaacgatcctgcagCTCTTCCTATGACATAGATGAGCACTGGGTAGACCCCG
TCCTGGTAACACTTCTCATGCACTAACAAGTTGGTTGCACCGGTGCTCCCAGGCGGGGGCCGTGGAGGGCAGGCGGACTAGGAGCCA
GC

: *TMPRSS2*, : *ERG*, **+1**: Transcription start, ctgcag: PstI

asC-31

+1gtgctcgcttcggcagcacatatactaacattggaacgatcctgcag**CTCATGCTCTTCCTATGACATAGATGAGCACTGGGT
AGACCCCGTCCTGGTAACACTCTTCATGCACTAACAAGTTGCTCCCAGGCGGGGGCCGTGGAGGGCAGGCGGACTAGGAGCCA
GCTTTGGG**

: *TMPRSS2*, : *ERG*, **+1**: Transcription start, ctgcag: PstI

asC-35

+1gtgctcgcttcggcagcacatatactaacattggaacgatcctgcag**TGAGCTCATGCTCTTCCTATGACATAGATGAGCACT
GGGTAGACCCCGTCCTGGTAACACTCTTCATGCACTAACCCCAGGCGGGGGCCGTGGAGGGCAGGCGGACTAGGAGCCAGCTT
TGGGGACC**

: *TMPRSS2*, : *ERG*, **+1**: Transcription start, ctgcag: PstI

asC-40

+1gtgctcgcttcggcagcacatatactaacattggaacgatcctgcag**TACGCTGAGCTCATGCTCTTCCTATGACATAGATGA
GCACTGGGTAGACCCCGTCCTGGTAACACTCTTCATGCAGCGGGGGCCGTGGAGGGCAGGCGGACTAGGAGCCAGCTTTGGGG
ACCCCGGG**

: *TMPRSS2*, : *ERG*, **+1**: Transcription start, ctgcag: PstI

asC-45

+1gtgctcgcttcggcagcacatatactaacattggaacgatcctgcag**TGTTATACGCTGAGCTCATGCTCTTCCTATGACATA
GATGAGCACTGGGTAGACCCCGTCCTGGTAACACTCTTC**GGCCGTGGAGGGCAGGCGGACTAGGAGCCAGCTTTGGGGACCCC
GGGGGACT

: *TMPRSS2*, : *ERG*, **+1**: Transcription start, ctgcag: PstI

asC-50

+1gtgctcgcttcggcagcacatatactaacattggaacgatc<u>ctgcag</u>GCTAATGTTATACGCTGAGCTCATGCTGTTCCTATGAC
ATAGATGAGCACTGGGTAGACCCCGTCCTGGTAACACTGGAGGGCAGGCGGACTAGGAGCCAGCTTTGGGGACCCCGGGGGACTCTC
TT

: *TMPRSS2*, : *ERG*, **+1**: Transcription start, ctgcag: PstI

asC-100

+1gtgctcgcttcggcagcacatatactaacattggaacgatc<u>ctgcag</u>TGGTTGCTGCATGGAAAGGGGACTAAGTAGCCAGGTCA
TCAGAGCCCTTTGCTAATGTTATACGCTGAGCTCATGTTCCACCAACTGGCTAGGGACTCGCAGGACCACCTGCCCCAACCTCGCGA
TG

: *TMPRSS2*, : *ERG*, **+1**: Transcription start, ctgcag: PstI

asD-2

+1gtgctcgcttcggcagcacatatactaacattggaacgatc<u>ctgcag</u>**<u>GAGACAGAAGAAAGATGAAGGGATAAGTGTCCAGAA</u>**
<u>TCCCTGGATCTGGGATGGAATAAAGGATCTGGATGGTAAGCACCCCGGGAGGCGCCCTGCCCGGCTGGCCCCAGCGCTCGACC</u>
<u>CTCGGGCG</u>

: *TMPRSS2*, : *ERG*, **+1**: Transcription start, ctgcag: PstI

asD-16

+1gtgctcgcttcggcagcacatatactaacattggaacgatc<u>ctgcag</u>GCTGGTAGAGGGAAGAGACAGAAGAAAGATGAAGGGAT
AAGTGTCCAGAATCCCTGGATCTGGGATGGAATAAAGGCCCTGCCCGGCTGGCCCCAGCGCTCGACCCTCGGGCGCACTCACCTGCC
GC

: *TMPRSS2*, : *ERG*, **+1**: Transcription start, ctgcag: PstI

asD-20

+1gtgctcgcttcggcagcacatatactaacattggaacgatcctgcagAGAGGCTGGTAGAGGGAAGAGACAGAAGAAAGATGA
AGGGATAAGTGTCCAGAATCCCTGGATCTGGGATGGAATTGCCCGGCTGGCCCCAGCGCTCGACCCTCGGGCGCACTCACCTG
CCGCGCCG

: *TMPRSS2*, : *ERG*, **+1**: Transcription start, ctgcag: PstI

asD-25

+1gtgctcgcttcggcagcacatatactaacattggaacgatcctgcagGAGAGAGAGGCTGGTAGAGGGAAGAGACAGAAGAAA
GATGAAGGGATAAGTGTCCAGAATCCCTGGATCTGGGATGGCTGGCCCCAGCGCTCGACCCTCGGGCGCACTCACCTGCCGCG
CCGCGCTC

: *TMPRSS2*, : *ERG*, **+1**: Transcription start, ctgcag: PstI

asD-31

+1gtgctcgcttcggcagcacatatactaacattggaacgatcctgcagGACAGAGAGAGAGAGGCTGGTAGAGGGAAGAGACAG
AAGAAAGATGAAGGGATAAGTGTCCAGAATCCCTGGATCCCCCAGCGCTCGACCCTCGGGCGCACTCACCTGCCGCGCCGCGC
TCCTCACA

: *TMPRSS2*, : *ERG*, **+1**: Transcription start, ctgcag: PstI

asD-35

+1gtgctcgcttcggcagcacatatactaacattggaacgatcctgcagGAGAGACAGAGAGAGAGAGGCTGGTAGAGGGAAGAGAC
AGAAGAAAGATGAAGGGATAAGTGTCCAGAATCCCTGAGCGCTCGACCCTCGGGCGCACTCACCTGCCGCGCCGCGCTCCTCACACC
CG

: *TMPRSS2*, : *ERG*, **+1**: Transcription start, ctgcag: PstI

asD-40

+1gtgctcgcttcggcagcacatatactaacattggaacgatcctgcagCTAGAGAGAGACAGAGAGAGAGAGGCTGGTAGAGGGAA
GAGACAGAAGAAAGATGAAGGGATAAGTGTCCAGAATTCGACCCTCGGGCGCACTCACCTGCCGCGCCGCGCTCCTCACACCCGCTT
TC

: *TMPRSS2*, : *ERG*, **+1**: Transcription start, ctgcag: PstI

asD-45

+1gtgctcgcttcggcagcacatatactaacattggaacgatcctgcagATCACCTAGAGAGAGACAGAGAGAGAGAGGCTGGTAGA
GGGAAGAGACAGAAGAAAGATGAAGGGATAAGTGTCCCCTCGGGCGCACTCACCTGCCGCGCCGCGCTCCTCACACCCGCTTTCACC
TC

: *TMPRSS2*, : *ERG*, **+1**: Transcription start, ctgcag: PstI

asD-50

+1gtgctcgcttcggcagcacatatactaacattggaacgatcctgcagATGGAATCACCTAGAGAGAGACAGAGAGAGAGAGGCTG
GTAGAGGGAAGAGACAGAAGAAAGATGAAGGGATAAGGGCGCACTCACCTGCCGCGCCGCGCTCCTCACACCCGCTTTCACCTCCGG
GC

: *TMPRSS2*, : *ERG*, **+1**: Transcription start, ctgcag: PstI

asD-100

+1gtgctcgcttcggcagcacatatactaacattggaacgatcctgcagAAGAAAAGTCACACGGTGGAGTGTGTTGGTGCCTGCTC
TTGCCATGAGAGATGGAATCACCTAGAGAGAGACAGAGCGGGGCAGGGGGCATCGGCGGGTCCCAGGCGCCCAGGTTCCCCTCCCCA
GC

: *TMPRSS2*, : *ERG*, **+1**: Transcription start, ctgcag: PstI

asB-35-100/100

+1gtgctcgcttcggcagcacatatactaacattggaacgatcctgcagAGGGATTAAAGGAACTGGTGTGCACTGGCCTGAGCCTT
GAAGAATGGGGTGTACTGGGTAAATCAAAATGGTGGGGAGCATTTCCAGATGGAGAAACTGCCTCACCTGCCGCGCTCCAGGCGGCG
CTCCCCGCCCCTCGCCCTCCGCCTCCGCCTCCGCCTCCTGCTTAGCTCGCGCCTACTCGGCCCGGCCCGCCCTGG

: *TMPRSS2*, : *ERG*, **+1**: Transcription start, ctgcag: PstI

asB-35-75/75

+1gtgctcgcttcggcagcacatatactaacattggaacgatc<u>ctgcag</u>TGGCCTGAGCCTTGAAGAATGGGGTGTACTGGGTAAAT
CAAAATGGTGGGGAGCATTTCCAGATGGAGAAACTGCCTCACCTGCCGCGCTCCAGGCGGCGCTCCCCGCCCCTCGCCCTCCGCCTC
CGCCTCCGCCTCCTGCTTAGCTCGC

: *TMPRSS2*, : *ERG*, **+1**: Transcription start, ctgcag: PstI

asB-35-50/50

+1gtgctcgcttcggcagcacatatactaacattggaacgatc<u>ctgcag</u>**<u>GTACTGGGTAAATCAAAATGGTGGGGAGCATTTCCA</u>**
<u>GATGGAGAAACTGCCTCACCTGCCGCGCTCCAGGCGGCGCTCCCCGCCCCTCGCCCTCCGCCTC</u>

: *TMPRSS2*, : *ERG*, **+1**: Transcription start, ctgcag: PstI

asB-35-30/30

+1gtgctcgcttcggcagcacatatactaacattggaacgatc<u>ctgcag</u>GTGGGGAGCATTTCCAGATGGAGAAACTGCCTCACCTG
CCGCGCTCCAGGCGGCGCTCCC

: *TMPRSS2*, : *ERG*, **+1**: Transcription start, ctgcag: PstI

asC-35-100/100

+1gtgctcgcttcggcagcacatatactaacattggaacgatc<u>ctgcag</u>AGAGCCCTTTGCTAATGTTATACGCTGAGCTCATGCTC
TTCCTATGACATAGATGAGCACTGGGTAGACCCCGTCCTGGTAACACTCTTCATGCACTAACCCCAGGCGGGGGCCGTGGAGGGCAG
GCGGACTAGGAGCCAGCTTTGGGGACCCCGGGGGACTCTCTTCCACCAACTGGCTAGGGACTCGCAGGACCACCT

: *TMPRSS2*, : *ERG*, **+1**: Transcription start, ctgcag: PstI

asC-35-75/75

+1gtgctcgcttcggcagcacatatactaacattggaacgatc<u>ctgcag</u>TGAGCTCATGCTCTTCCTATGACATAGATGAGCACTGG
GTAGACCCCGTCCTGGTAACACTCTTCATGCACTAACCCCAGGCGGGGGCCGTGGAGGGCAGGCGGACTAGGAGCCAGCTTTGGGGA
CCCCGGGGGACTCTCTTCCACCAAC

: *TMPRSS2*, : *ERG*, **+1**: Transcription start, ctgcag: PstI

asC-35-50/50

+1gtgctcgcttcggcagcacatatactaacattggaacgatcctgcagAGATGAGCACTGGGTAGACCCCGTCCTGGTAACACTCT
TCATGCACTAACCCCAGGCGGGGGCCGTGGAGGGCAGGCGGACTAGGAGCCAGCTTTGGGGA

: *TMPRSS2*, : *ERG*, **+1**: Transcription start, ctgcag: PstI

asC-35-30/30

+1gtgctcgcttcggcagcacatatactaacattggaacgatcctgcagCCGTCCTGGTAACACTCTTCATGCACTAACCCCAGGCG
GGGGCCGTGGAGGGCAGGCGGA

: *TMPRSS2*, : *ERG*, **+1**: Transcription start, ctgcag: PstI

asD-35-100/100

+1gtgctcgcttcggcagcacatatactaacattggaacgatcctgcagGCCATGAGAGATGGAATCACCTAGAGAGAGACAGAGAG
AGAGAGGCTGGTAGAGGGAAGAGACAGAAGAAAGATGAAGGGATAAGTGTCCAGAATCCCTGAGCGCTCGACCCTCGGGCGCACTCA
CCTGCCGCGCCGCGCTCCTCACACCCGCTTTCACCTCCGGGCGGGGCAGGGGGCATCGGCGGGTCCCAGGCGCCC

: *TMPRSS2*, : *ERG*, **+1**: Transcription start, ctgcag: PstI

asD-35-75/75

+1gtgctcgcttcggcagcacatatactaacattggaacgatcctgcagGAGAGACAGAGAGAGAGAGGCTGGTAGAGGGAAGAGAC
AGAAGAAAGATGAAGGGATAAGTGTCCAGAATCCCTGAGCGCTCGACCCTCGGGCGCACTCACCTGCCGCGCCGCGCTCCTCACACC
CGCTTTCACCTCCGGGCGGGGCAGG

: *TMPRSS2*, : *ERG*, **+1**: Transcription start, ctgcag: PstI

asD-35-50/50

+1gtgctcgcttcggcagcacatatactaacattggaacgatc<u>ctgcag</u>AGAGGGAAGAGACAGAAGAAAGATGAAGGGATAAGTGT
CCAGAATCCCTGAGCGCTCGACCCTCGGGCGCACTCACCTGCCGCGCCGCGCTCCTCACACC

: *TMPRSS2*, : *ERG*, **+1**: Transcription start, ctgcag: PstI

asD-35-30/30

+1gtgctcgcttcggcagcacatatactaacattggaacgatc<u>ctgcag</u>AGATGAAGGGATAAGTGTCCAGAATCCCTGAGCGCTCG
ACCCTCGGGCGCACTCACCTGC

: *TMPRSS2*, : *ERG*, **+1**: Transcription start, ctgcag: PstI
